# Environmental Influences on Patterns of Vertical Movement and Site Fidelity of Grey Reef Sharks (*Carcharhinus amblyrhynchos*) at Aggregation Sites

**DOI:** 10.1371/journal.pone.0060331

**Published:** 2013-04-10

**Authors:** Gabriel M. S. Vianna, Mark G. Meekan, Jessica J. Meeuwig, Conrad W. Speed

**Affiliations:** 1 Australian Institute of Marine Science, Perth, Western Australia, Australia; 2 Oceans Institute and School of Animal Biology, University of Western Australia, Perth, Western Australia, Australia; The Australian National University, Australia

## Abstract

We used acoustic telemetry to describe the patterns of vertical movement, site fidelity and residency of grey reef sharks (*Carcharhinus amblyrhynchos*) on the outer slope of coral reefs in Palau, Micronesia, over a period of two years and nine months. We tagged 39 sharks (mostly adult females) of which 31 were detected regularly throughout the study. Sharks displayed strong inter-annual residency with greater attendance at monitored sites during summer than winter months. More individuals were detected during the day than at night. Mean depths of tagged sharks increased from 35 m in winter to 60 m in spring following an increase in water temperature at 60 m, with maximum mean depths attained when water temperatures at 60 m stabilised around 29°C. Sharks descended to greater depths and used a wider range of depths around the time of the full moon. There were also crepuscular cycles in mean depth, with sharks moving into shallower waters at dawn and dusk each day. We suggest that daily, lunar and seasonal cycles in vertical movement and residency are strategies for optimising both energetic budgets and foraging behaviour. Cyclical patterns of movement in response to environmental variables might affect the susceptibility of reef sharks to fishing, a consideration that should be taken into account in the implementation of conservation strategies.

## Introduction

Free-ranging marine predators such as sharks live in a three-dimensional environment where they are able to move in both horizontal and vertical planes. In coral reef ecosystems, most studies of the movement of sharks have focused on defining patterns of use of space on a horizontal plane, many with the ultimate goal of contributing to spatial management strategies, such as marine protected areas, to ensure the adequate conservation of shark populations. Such studies show that site fidelity is a common phenomenon in many species, including whitetip (*Triaenodon obesus*), tawny nurse (*Ginglymostoma cirratum*), blacktip (*Carcharhinus melanopterus*), Caribbean (*C. perezi*) and grey reef (*C. amblyrhynchos*) sharks [1], [2], [3], [4], [5], [6]. The degree of fidelity appears to vary according to life history stage, availability of resources and area of suitable habitat [6], [7], [8]. Strong site fidelity of juveniles to nursery areas is evident in lemon (*Negaprion brevirostris*), blacktip and Caribbean reef sharks and is thought to be due to the advantages of nurseries in terms of predator avoidance and food availability [9], [10], [11]. Site fidelity is also common in adult reef sharks, although typically more sporadic when compared to juveniles, which might be partially explained by ontogenetic increases in the size of home ranges [8], [12]. Adult site fidelity is argued to be advantageous for a number of reasons, including mating, feeding, pupping and resting [12].

While these studies have contributed to our understanding of the habitat preferences of sharks in reef ecosystems, there is an almost complete lack of equivalent data on the movements of reef sharks in the vertical plane of the water column. In the open ocean, cycles in vertical movement are a fundamental part of the behaviour of predatory species that reflect both changes in physical environments and distributions of prey. For example, pelagic species including swordfish (*Xiphias gladius*), yellowfin (*Thunnus albacares*) and big eye (*T.obesus*) tunas and mako sharks (*Isurus oxyrinchus*) display diel vertical migrations, where they descend to deep water during the day and remain in relatively shallow water at night, a pattern that is thought to follow cycles in the distribution of prey [13], [14], [15], [16]. In temperate systems, some coastal species, such as the leopard shark (*Triakis semifasciata*), also show daily vertical migrations and actively use shallow, warm waters in the day and late afternoon to increase the core body temperature to optimise rates of digestion, growth and gestation [17].

The limited information that is available suggests that cycles in vertical movement are also a feature of the behaviour of reef sharks. For example, similar to leopard sharks, grey reef and blacktip reef sharks aggregate in shallow warm waters of sand flats in the afternoon possibly to increase growth and gestation rates [7], [18], while short-term (up to 20 days) tracking suggests that Caribbean reef sharks have a preference for shallow water (<40 m) during the night [19]. Whitetip reef sharks do not appear to display diel patterns in depth preferences, but occupy a wider depth range during the night when actively hunting than during the day when resting [4], [20]. Together, these studies suggest a range in patterns of vertical movements by sharks in coral reefs that reflect a variety of ecological drivers.

A better understanding of the ecology of reef sharks in coral reef systems requires the examination of movement and residency patterns on both horizontal and vertical planes. Here, we describe spatial and temporal patterns in the vertical movements and residency of the grey reef shark, one of the most common and abundant sharks on coral reefs across the Indo-Pacific. At our study site in Palau, Micronesia, grey reef sharks tend to form predictable aggregations on outer parts of reef slopes and crests exposed to high current flow. We used acoustic telemetry to describe patterns of spatial and temporal use of aggregation sites by grey reef sharks over multiple years. A combination of acoustic telemetry and environmental data was also used to test the hypothesis that the vertical movements and residency patterns by grey reef sharks were related to environmental variables, notably water temperature. Our study contributes to a better understanding of the ecology of these animals and has implications for the management of sharks at aggregation sites, an important driver for diving ecotourism and the Palauan economy [21].

## Methods

### Ethics statement

This project was conducted under the Republic of Palau Marine Research Permit no. RE-09-26 and the Koror State Marine Research Permit no. 10–204. Shark tagging in 2011 was also conducted under UWA animal ethics permit no. RA/3/100/975, in adherence to provisions contained within the Australian Code of Practice for the Care and Use of Animals for Scientific Purposes.

### Study location

**Figure 1:**
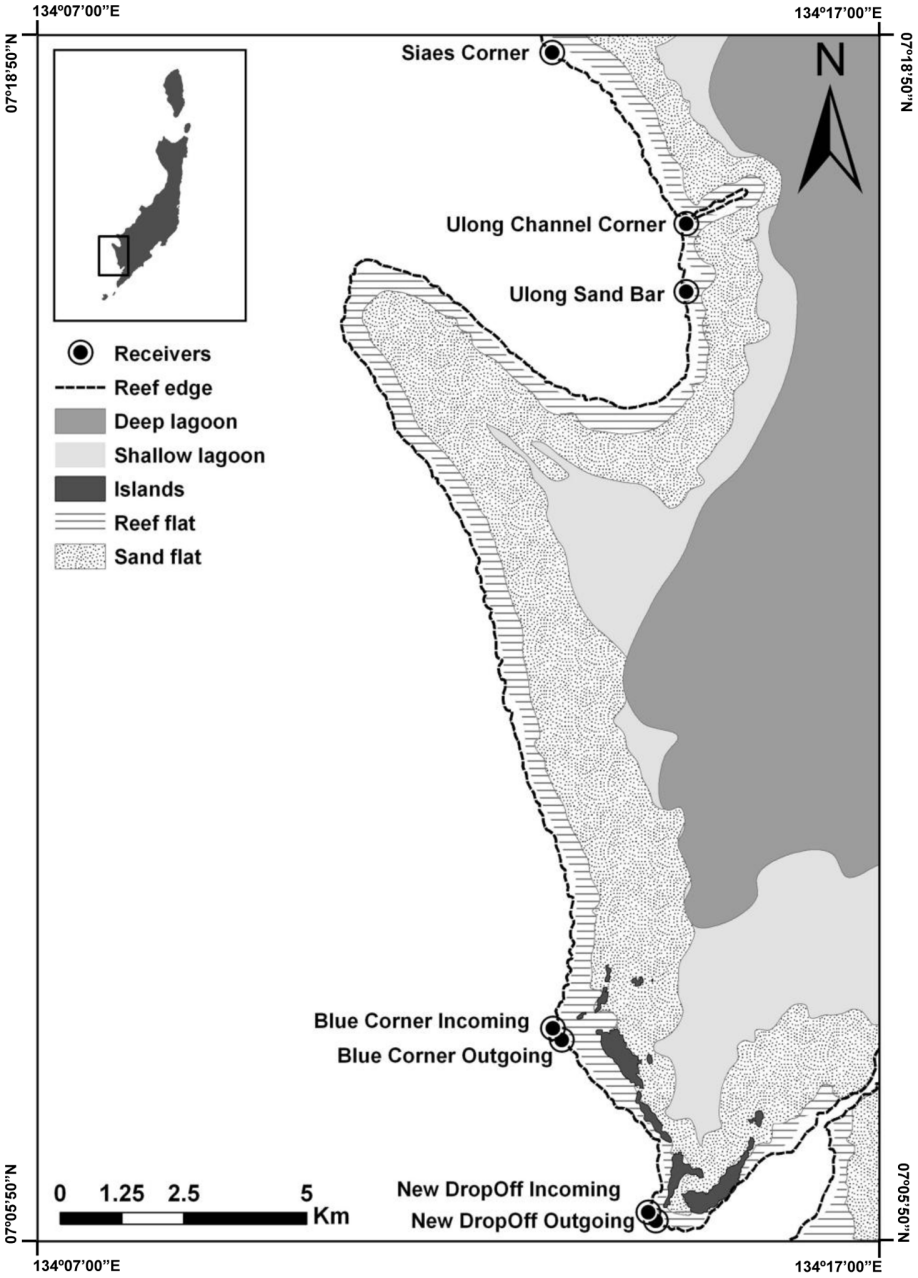
Study area in Palau. Outer reef slope of the southwest barrier reef of Palau, showing location of receivers. Top left box indicates the study site in the main island platform. The “shallow lagoon” shade represents depths down to 5 m, while “deep lagoon” areas might reach depths of 20 m.

Palau is an archipelago of approximately 300 islands and atolls in the northwest Pacific (7°N, 134°W). Our study location was the edge of the main island platform that consists of a large shallow-water lagoon arrayed with small, uplifted limestone islands and a large volcanic island, all of which are enclosed by a 260 km barrier reef [22]. Grey reef sharks regularly aggregate at sites along the outer reef slope in the southwest (leeward) quadrant of the barrier reef (Figure 1) at promontories where the crenulated reef margin juts out into the flow of the prevailing current [21].

### Acoustic array and shark tagging

We used acoustic receivers (VR2w, Vemco) to monitor the attendance of tagged sharks at five aggregation sites. We moored receivers at depths between 25 and 40 m on the reef wall or slope and downloaded data from them at one to eight month intervals. The acoustic array monitored two areas on the barrier reef characterised by vertical walls and steep slopes [22]. The receivers were distributed over a linear distance of approximately 6 km in the northern area and 5 km in the southern area (Figure 1). The first receiver was deployed in November 2008, with the remainder deployed between May and July 2009.

We used hand reels fitted with baited barbless hooks to catch sharks at each of the receiver deployment sites within an area (Figure 1, Table 1). Once caught, sharks were brought alongside the boat and restrained within a canvas stretcher, which was then lifted onboard. Sharks were turned upside-down to induce tonic immobility and placed in a holding tank with a constant flow of water into the mouth and through the gills. We recorded the sex, measured the total length (L_T_) and surgically implanted an acoustic transmitter into the peritoneal cavity of each shark [23]. This tagging procedure typically required less than ten minutes from the moment the shark was caught to the moment it was released. We classified individuals as sexually mature according to the L_T_ [24]. We used a combination of Vemco V16-5H coded tags (power output 165 dB, frequency of 69 Khz) with an estimated battery life of 3.4 years in 2008 and 2009 and V16-6H coded tags (power output 160 dB, frequency of 69 Khz) with an estimated battery life of 10 years in 2011. Ten of these tags were also fitted with pressure sensors that recorded depths to a maximum of 136 m (five V16-5H, deployed in 2008), 204 m (two V16-6H deployed in 2011) or 304 m (three V16-6H deployed in 2011).

**Table 1:** Residency index and mean number of hours (±SE) grey reef sharks were detected at each monitored site daily (brackets).

Shark			Northern area			Southern area			
Tag no.	Gender	L_T_ (cm)	Siaes Corner	Ulong Channel	Ulong Sand Bar	Blue Corner in	Blue Corner out	New Drop-off in	New Drop-off out
14506	Female	148	0	0	0	5% (4±5.3)	4% (3±2.2)	60% (4±2.6)	67% (7±3.9)*
14507	Female	145	0	0*	100% (19±5.1)	0	0	0	0
14508	Female	126	0	0	0	29% (3±1.9)	74% (22±2.7)*	0	0
14509	Female	144	0	0	0	16% (2±1.1)*	0	0	0
14510	Female	153	2% (1±0.6)	4% (13±6.8)*	39% (7±5.5)	0	0	0	0
15764	Female	146	2% (5±4.1)	100% (13±5.4)*	0	0	0	0	0
15765	Female	144	4% (3±1.9)	100% (9±5.2)*	0	0	0	0	0
15766	Female	145	4% (2±0.8)	85% (3±2.6)*	0	0	0	0	0
15767	Female	133	6% (4±1.8)	96% (22±3.4)*	0	0	0	0	0
15768	Female	140	0	100% (22±2.4)*	0	0	0	0	0
32535	Female	144	0	0	0	5% (4±3.7)	4% (3±2.2)	44% (3±2.1)	48% (21±4.2)*
32536	Female	134	0	0	0	20% (3±3.2)	96% (22±3)*	0	0
32537	Female	130	0	0	0	5% (9±5.7)	4% (5±2.6)	59% (14±5.3)*	46% (4±2.3)
32538	Female	123	0	0	0	73% (3±2)	96% (17±3.8)*	4% (3±1.9)	4% (2±0.9)
32540	Female	130	0	0	0	23% (2±1.5)	96% (22±2.7)*	0	0
32542	Female	141	0	0	0	5% (9±7.3)	4% (2±1.3)	56% (11±3.5)*	50% (8±4.8)
32543	Female	154	0	0	0	42% (4±3.7)	96% (17±4.8)*	0	0
53362	Female	134	0	0	0	61% (21±3.6)*	41% (4±2.5)	3% (4±2.3)	4% (3±1.6)
53365	Female	147	5% (5±5.9)	98% (23±3.3)*	1% (2±0.8)	0	0	0	0
53366	Female	144	0	0	0	28% (4±3.4)*	23% (4±2.6)	34% (4±2.8)	62% (6±4.6)
53367**	Female	139	0	1% (1±0)	0	21% (2±2.8)	89% (23±2.6)	1% (2±2.7)	1% (2±2.1)
53368	Female	139	7% (3±2.3)	98% (21±3.6)*	1% (2±1.1)	0	0	0	0
53369**	Female	145	0	0	0	10% (4±3.5)	89% (22±2.8)	0	0
53370	Female	155	0	0	0	71% (21±3.3)*	62% (5±3.6)*	3% (3±1.7)	3% (2±1.5)
53372	Male	126	80% (6±3.7)*	1% (4±3)	0	0	0	0	0
53373	Female	158	33% (5±4)*	0% (3±1.3)	0	0	0	0	0
53375	Female	157	76% (5±2.9)*	6% (6±6.9)	0	0	0	0	0
53388	Female	146	0	0	0	58% (7±3.5)	72% (7±3.6)*	0	0
53389	Female	149	0	0*	0	0	52% (7±4.7)	0*	0
53390	Female	139	4% (4±2.7)	98% (12±5.1)*	0	0	0	0	0
53391	Female	146	0	0	0	10% (3±2.6)	12% (3±2)	74% (5±3.1)	77% (21±4.5)*
53392	Female	145	16% (4±2.2)	59% (5±4.2)*	93% (9±4.6)	1% (1±0)	2% (1±0)	1% (1±0)	1% (1±0)
53393	Female	123	0	0	0	51% (4±2.3)	86% (14±5.1)*	0	0
53395	Female	143	85% (5±3.3)	27% (6±4.9)*	2% (2±0.7)	0	0	0	0
53396	Female	155	0	0	0	69% (22±3.5)*	40% (3±2.1)	3% (3±2.2)	3% (3±1)
53397	Female	125	2% (3±1.8)	81% (11±8.1)*	0	0	0	0	0

(*) indicates the site where each individual was tagged, (**) indicates sharks tagged 300 m north of Blue Corner. L_T_ indicates total length of individual.

We tagged a total of 39 grey reef sharks during November 2008 (*n* = 8), May 2009 (*n* = 18) and March 2011 (*n* = 13). Tagged sharks included 34 adult females (mean L_T_ = 142±11 cm), four sub-adult females (mean L_T_ = 124±1 cm) and one sub-adult male (L_T_ = 126 cm). Of these, 17 sharks were tagged in the northern area and 22 in the southern area. Two of the tagged sharks were not detected by the array and one individual was detected for only seven days; data for these sharks were not included in analyses.

In April 2011, we conducted range testing of the receivers in the northern site by deploying a test tag (V16-6H, power output 160 dB, frequency of 69 Khz, fixed delay) and estimating the detection coefficient at intervals of 200 m along transects parallel and perpendicular to the receiver deployment sites. The long-term performance of the receivers was of concern given the large number of tagged individuals in an environment with a complex current regime and reef habitat [22]. In order to assess performance we used metrics developed by Simpfendorfer et al. (2008) to analyse: (1) code detection efficiency, which provided information on the percentage of tagged animals that had valid detections (consisting of a complete code sequence) and (2) rejection coefficient, which provided an estimate of rejected detections due to incomplete codes detected by the receivers [25]. To estimate levels of biotic and abiotic interference in detection probabilities [26], we deployed a control tag on the reef wall in the southern area for a period of 141 days. This tag was located 200 m from the receiver at Blue Corner Incoming (Figure 1).

### Data analysis

We used hourly and daily attendance as metrics to describe the general patterns of site fidelity of sharks at deployment sites of receivers. A shark was considered to be present if two or more detections were recorded in the same day. The use of metrics based on hourly or daily attendance (instead of detections) reduced the effects of differences in detection probability related to the use of tags with different signal outputs. To describe site fidelity, we estimated the residency index as the proportion of monitored days during which a shark attended a given site. We also estimated the mean number of hours detected per day when a shark attended a given site. We classified a shark as “resident” at a site if it had a residency index higher than 0.5 and the mean number of hours detected per day was equal or higher than 12 (i.e., 50% of the total hours available in a day). We considered sharks as inter-annual residents when an animal had an annual residency index equal or higher than 0.5 over consecutive years. We also calculated the daily attendance index as the longest time series of consecutive days each shark attended a monitored site divided by the total number of days the shark was monitored. As time series were often interrupted by downloading of receivers, each portion of the interrupted series was considered to be independent and for this reason, the daily attendance index was likely to be a conservative metric of site fidelity at monitored sites.

We quantified differences in site preferences by calculating the standardised daily attendance as the percentage of sharks tagged in each area attending each receiver on each day. We used ANOVA and a t-test [27] to compare site preferences in the southern and northern areas respectively. To determine movement between these areas, we estimated the minimum linear dispersal [3], minimum dispersal time (as the time between the last detection in the residency area and the time of the first detection in the visiting area), and time spent (hours detected) in each visiting event. A shark was considered to be present in the visited area if two or more detections were recorded by the receivers within a period of two hours. For all metrics, mean values and standard deviations (±SD) are reported.

To analyse diel patterns in reef attendance we applied a Fast-Fourier transformation [28] to the detection frequency of each shark when the individual had a residency index higher than 0.5 [29]. The hourly detection frequencies were corrected to account for variations in the detection probability [26]. We analysed the northern and southern areas separately, due to preliminary results suggesting that there was limited movement away from the area in which each animal was tagged. We also calculated mean detection frequency of sharks combined per month in each area and employed circular regression to quantify seasonal patterns in attendance [30]. We corrected the detection frequencies using the correction factors calculated from the data of our control tag [26].

We applied a generalised linear model (GLM) with bootstrap sampling to examine the effects of environmental factors on the patterns of depth usage of sharks in 2010, using the mean daily depth of all tagged sharks as the response variable. For this model, water temperature and moon phase were used as explanatory variables. Our temperature dataset consisted of mean weekly water temperature at 57 m depth in the proximity of the monitored sites in both areas (source: Coral Reef Research Foundation, Palau). There was little variation in the temperature between the northern and southern areas, thus we combined data from both for subsequent analyses. We classified the moon phases according to luminosity, where “new” phases had <10% illumination, “half” phases had 11–90% illumination and “full” phases >90% illumination [31]. Percentage of illumination was obtained from United States Naval Observatory Astronomical Applications Department (USA Astronomical Application Department website. Available: http://aa.usno.navy.mil/data/docs/MoonFraction.php. Accessed 2012 March 3). We also used circular regression to identify patterns of depth usage in relation to diel cycles. As circular regression has low sensitivity to missing data [30], we used the mean depth of the sharks combined per hourly bin over the entire study period for the analysis.

We also used GLMs to establish the relationship between shark attendance and environmental variables within each area. The total number of individual sharks present per hour was the response variable, with tide phase (Tide), month (Month) and time of day (Day/Night; day defined as between 6 am and 6 pm) as the explanatory variables. High and low tide phases were defined as one hour prior to and following the slack tide [32].

Instantaneous records of shark attendance were aggregated into hourly estimates using a subset function in R [33] that selected 500 values from the data record for each shark. Due to the autocorrelation inherent in the data, the assumption of temporal independence was violated [34]; we addressed this violation by using a matched-block sampling with replacement technique [35], [36]. Briefly, this method sub-samples and replaces optimum block lengths from the dataset that maintain some of the autocorrelation structure. Blocks were then joined in a random order to create the uncorrelated bootstrapped sample [35], [36], [37]. We then applied the model-fitting process to 100 bootstrapped samples and used the median and 95% bootstrapped confidence intervals (2.5 and 97.5 percentiles) of the small sample-corrected Akaike's information criterion [38] test statistics: AIC*_c_*, ΔAIC*_c_* (difference between AIC*_c_* of a given model to the model of best fit), *w*AIC*_c_* (AIC*_c_* weight) and percent deviance explained (%DE) to rank and weight models.

## Results

### Receiver performance

Our array of receivers operated continuously during the period of study however, due to technical issues, the receivers from the Blue Corner Incoming and Blue Corner Outgoing sites (Figure 1) were not operational from April to November 2010 and March to April 2011, respectively (Figure S1). Range testing indicated that there was an overall decrease in the detection coefficient within a 200 m radius of the receivers. All receivers (with the exception of the receiver at Ulong Sand Bar) operated with overall mean code detection efficiency (CDE) above 0.4 for most of the period of the study (Figure S2). Following the last deployment of tags in April 2011, there was a considerable decrease in CDE for a number of receivers in both the northern and southern areas. A concurrent increase in the rejection coefficient values (RC) suggests that tag collisions likely contributed to the drop in performance of receivers at this time. We found no cyclical variation of the hourly detection frequencies of the control tag (R^2^ = 0.13, p = 0.07), however, the daily detection frequency presented a weak 29-day cycle (R^2^ = 0.17, p = 0.02).

### General attendance/residency

The receivers recorded a total of 2.3 million detections of 37 sharks over a period of 33 months. Of these, 31 (84%) sharks were detected for 70% to 100% of the monitored weeks. Of the remaining sharks, four were detected daily or weekly for two to 21 months following tagging, although after this time detections ceased. One adult female that was detected at sites on a weekly basis for 14 months after tagging was then not detected for 12 months, after which time she returned to the receiver array and was detected daily for the following two months until the final data download (Table 1).

On average, tagged sharks were monitored for 594±370 days (Table 2). Twenty individuals (55%) were classified as residents of a given monitored site (Table 1). Overall the residency index among the tagged sharks was 0.8±0.2, with a mean daily attendance index of 0.4±0.3 (Table 1 and 2). Seventeen of the 26 sharks (65%) tagged in 2008 and 2009 displayed inter-annual residency. On average, individuals were detected for 14±3 hours per day, suggesting that although individuals could have exited the array several times they remained in the vicinity of receivers for extended periods during the day.

**Table 2:** Attendance metrics of grey reef sharks tagged in Palau.

Attendance metrics (*n* = 37)	Mean ± SD	Min	Max
Number of days monitored	594±370	13	1114
Number of days detected	483±314	7	910
Maximum number of days continuously detected	191±97	4	343
Residency index	0.8±0.2	0.5	1.0
Daily attendance index	0.4±0.3	0.0	1.0
Mean number of hours detected per day	14±3	1	23

Most sharks were detected regularly at sites adjacent to where they were tagged (Table 1). Movement between the northern and southern areas was low and recorded for only four sharks. Of these, two individuals were recorded twice out of the area where they were tagged, while the remaining two sharks attended their non-residency area only once. The mean minimum linear distance of movements of these animals was 17.2±2.1 km and the minimum dispersal time ranged from 10 to 53 hours, but averaged around 13 hours. Attendance time was typically short as most sharks were detected at their non-residency areas for a maximum of four hours. The only male shark tagged by the study was detected in its non-residency area for nine successive hours.

**Figure 2:**
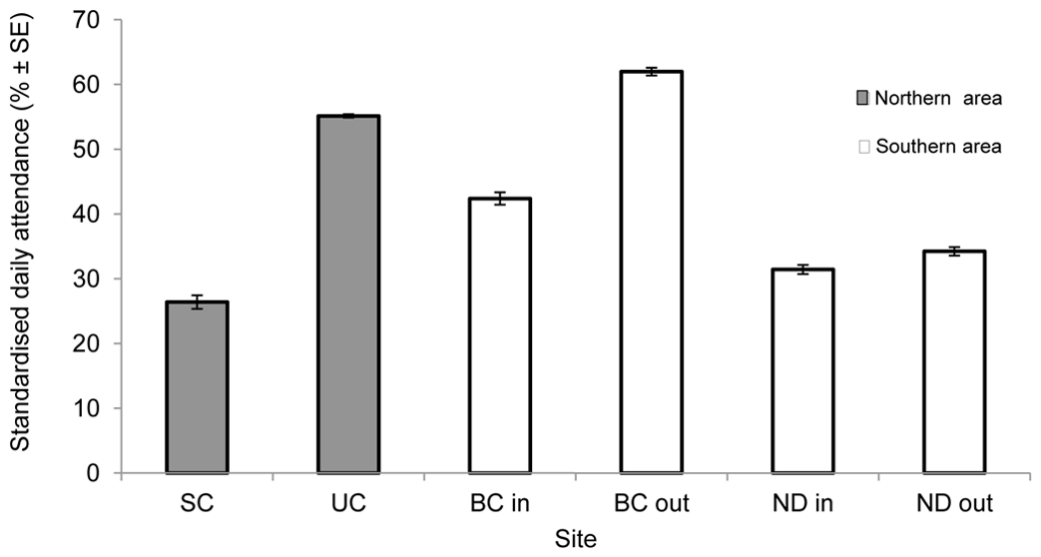
Standardised mean daily attendance of grey reef sharks in the monitored areas in Palau. Legends represent receivers at monitored site: SC =  Siaes Corner, UC = Ulong Channel, BC in =  Blue Corner Incoming, BC out =  Blue Corner Outgoing, ND in =  New Drop-off Incoming and ND out =  New Drop-off Outgoing. Ulong Sand Bar receiver is not included.

There were significant differences in the standardised daily attendance of sites within each area (t-test northern area: t = −26.7, p<0.01; ANOVA southern area, F = 170.6, p<0.01), with Ulong Channel (northern area) and Blue Corner Outgoing (southern area) having higher attendance of sharks than the other sites within the respective area (Figure 2).

**Figure 3:**
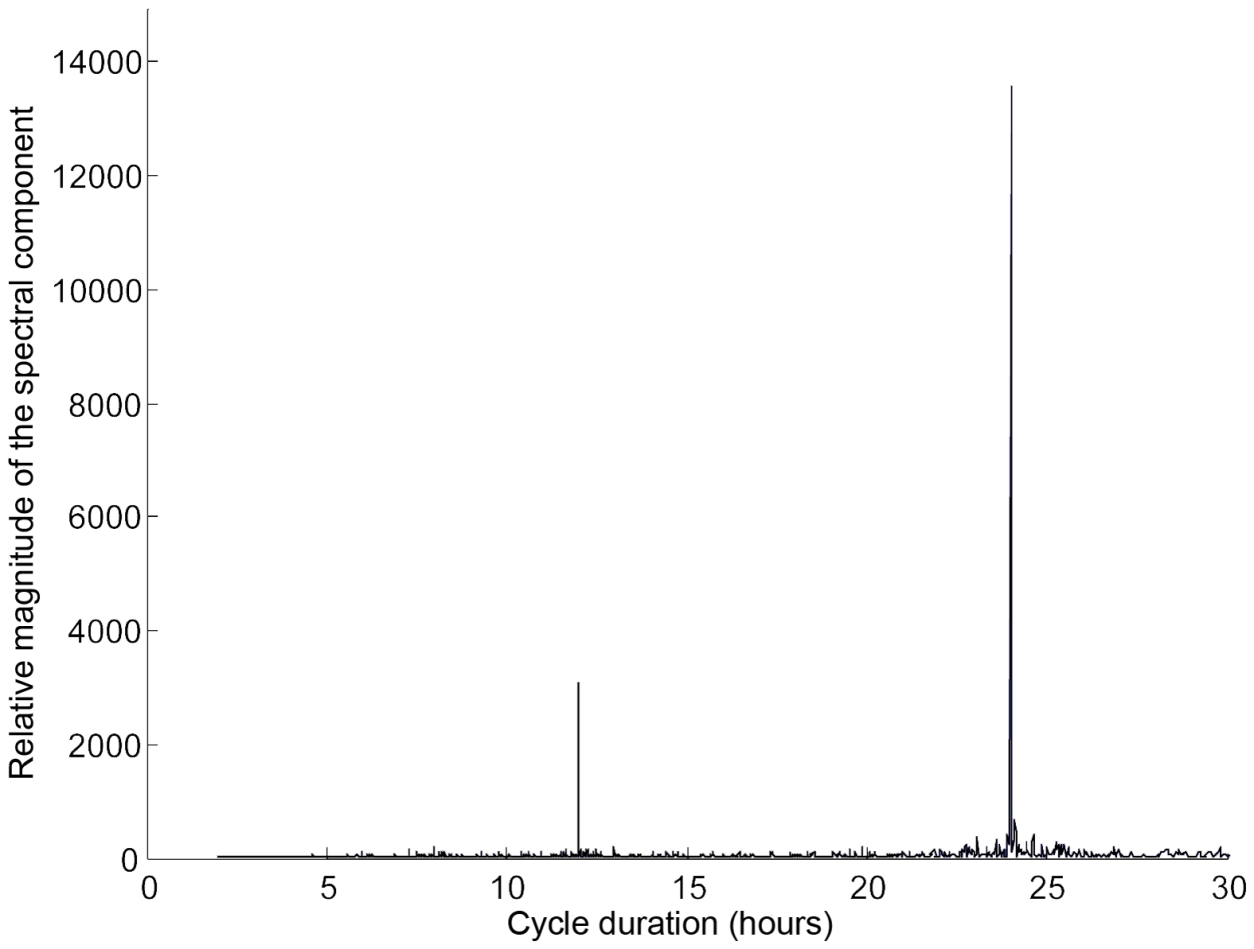
Fast-Fourier transformation of hourly detection frequencies of a grey reef shark in Palau. Diel patterns of corrected detection frequencies are represented as peaks of relative magnitude of spectral component. The transformation shows the diel periodicity of detection frequencies of a female grey reef shark (no. 53366, L_T_ = 144 cm), a representative example of diel cycles of detection frequencies of the sharks tagged in Palau.

**Figure 4:**
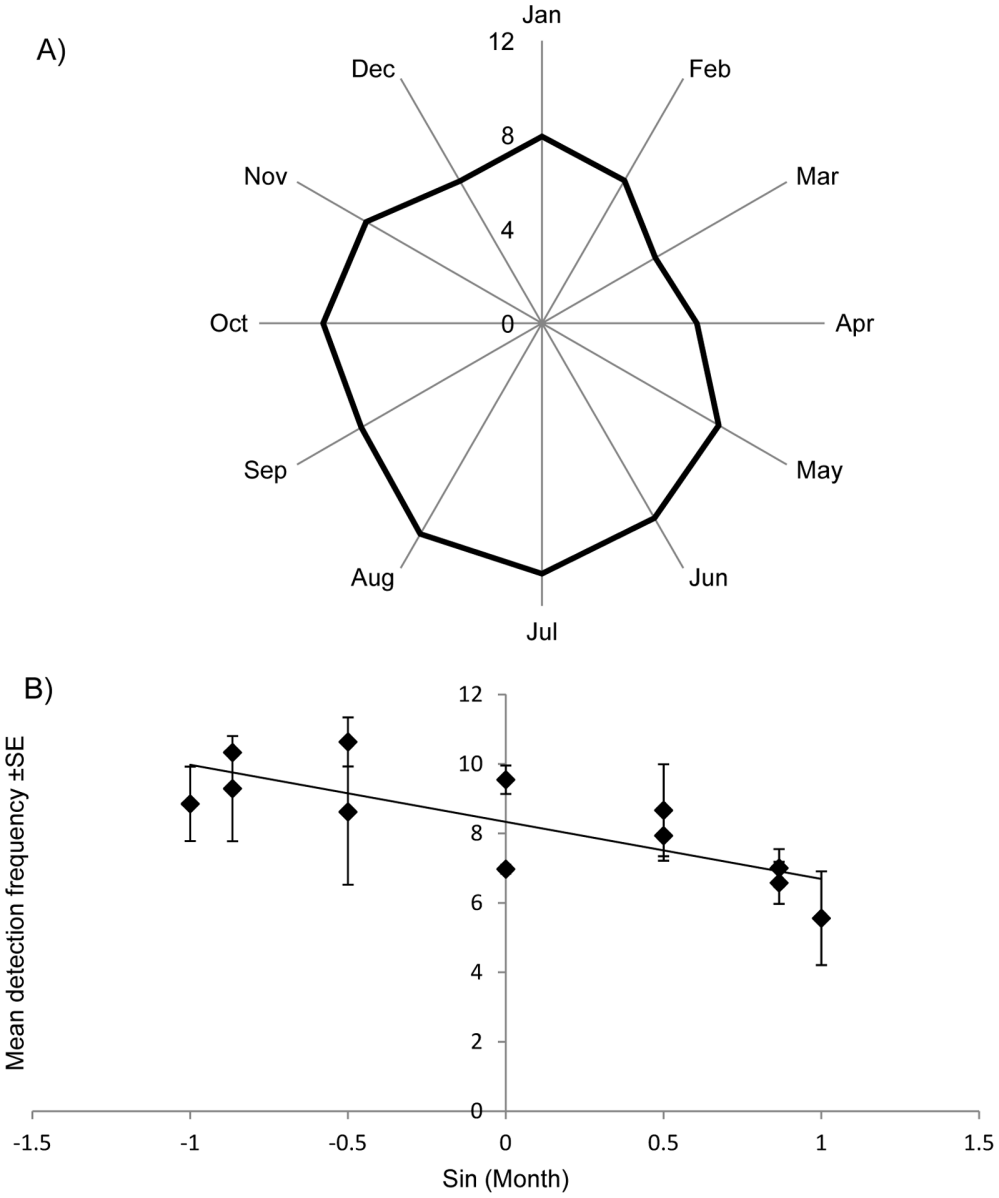
Mean detection frequencies of grey reef sharks per month in Palau. A) Polar plot of monthly mean daily detection frequency. Months are transformed and expressed as angles, mean daily detection frequencies in a given month (y-axis, areas combined) are represented as distance from the origin. Detection frequencies were corrected by the detection probabilities in each month, calculated from data of a control tag. B) Linear regression representing the mean daily detection frequency per month (areas combined) as a function of sin-transformed months. Equation y = −1.64x+8.33, R^2^ = 0.60.

All individuals in both areas showed strong 24 hour cycles in detection frequency (Figure 3). A smaller, 12 hour peak was also evident for two thirds of the sharks in the northern area and almost all (88%) of the sharks in the southern area. We also found significant differences in the mean daily detection frequencies per month for all sharks (Table 3), indicating that although sharks visited the monitored areas regularly through the year, there was a degree of seasonality, with a higher detection frequencies recorded mainly during summer (June to September) and lower detection frequencies in winter and spring (January to April) (Figure 4).

**Table 3:** Summary output of linear regressions of monthly mean detection frequency circular transformed.

Area	n	p-value	R^2^	SEE	Intercept	SE int	Slope	SE slope
Northern	12	0.0002	0.8	1.0	8.32	0.3	−2.54	0.4
Southern	12	0.017	0.5	1.4	8.17	0.0	−1.74	0.6
Mean	12	0.002	0.6	1.0	8.33	0.3	−1.64	0.0

“Mean” represents mean value of northern and southern areas. SEE =  standard error of estimate for the model (liner regression), SE int =  standard error of the intercept, SE slope =  standard error of slope.

**Figure 5:**
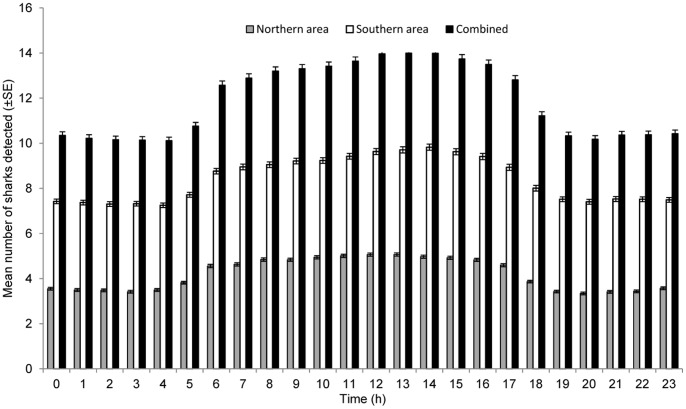
Hourly attendance patterns of grey reef sharks at monitored sites in Palau. Mean number of sharks detected in each hourly bin throughout the study period.

The GLM analysis indicated that a combination of daily and tidal factors influenced the pattern of reef attendance by sharks (Table 4), with more individuals attending the monitored sites during the daytime (Figure 5) and at low tide. The top-ranked model for the northern area (*w*AIC*_c_*  = 0.98) included these two variables with an interaction and had the best goodness-of-fit, explaining 19.8% of the deviance in the data. In the southern area, the model that provided the top-ranked fit (*w*AIC*_c_*  = 0.43) included Day/Night and Tide as covariates (Table 4) and explained 11.6% of the deviance in the data. In both areas, the amount of deviance explained by Tide was small (0.16% in the north and 0.36% in the south), indicating a greater effect of the daily cycle on the presence of sharks at sites within areas.

**Table 4:** Generalised Linear Models ranking results of number of grey reef sharks detected per hourly bin (Indivis as response variable) versus the following explanatory variables: months (Month), phase of the diel cycle (Day/Night), phase of the tidal cycle (Low, Incoming, High, Outgoing) (Tide).

Area	Model	LL	df	AICc	dAIC*c*	*w*AIC*c*	%DE
**Northern**	Indivs∼1 (Null)	−16329.73	1	32661.46	1263.462	0	0
	Indivs∼Month	−16222.97	2	32449.94	1051.948	0	3.3079
	Indivs∼Day/Night	−15703.06	2	31410.12	12.123	0.0023	19.4174
	Indivs∼Tide	−16324.45	4	32656.9	1258.908	0	0.1636
	Indivs∼Day/Night+Tide	−15698.53	5	31407.07	9.077	0.0106	19.5576
	Indivs∼Day/Night*Tide	**−15690.99**	**8**	**31397.99**	**0**	**0.9871**	**19.7913**
	Indivs∼Tide+Month	−16217.77	5	32445.55	1047.555	0	3.469
	Indivs∼Tide*Month	−16214.47	8	32444.96	1046.963	0	3.5713
**Southern**	Indivs∼1 (Null)	−14064.56	1	28131.11	261.630	0.0000	0.0000
	Indivs∼Month	−14023.44	2	28050.88	181.400	0.0000	3.5262
	Indivs∼Day/Night	−13933.11	2	27870.22	0.742	0.2990	11.2731
	Indivs∼Tide	−14060.32	4	28128.65	259.171	0.0000	0.3630
	Indivs∼Day/Night+Tide	−13929.74	5	27869.48	0.000	0.4333	11.5625
	Indivs∼Day/Night*Tide	**−13927.21**	**8**	**27870.45**	**0.963**	**0.2677**	**11.7791**
	Indivs∼Tide+Month	−14019.37	5	28048.76	179.272	0.0000	3.8750
	Indivs∼Tide*Month	−14018.46	8	28052.94	183.458	0.0000	3.9534

Models compared based on Akaike's Information Criteria corrected for small samples (AICc). LL: Maximum Log Likelihood, df: degrees of freedom, dAICc: difference of AICc of a given model to the model with best fit, wAICc: AICc weight and %DE: percentage of deviance explained. Model with best fit highlighted (bold). (*) Interaction between variables.

### Vertical movements

**Figure 6:**
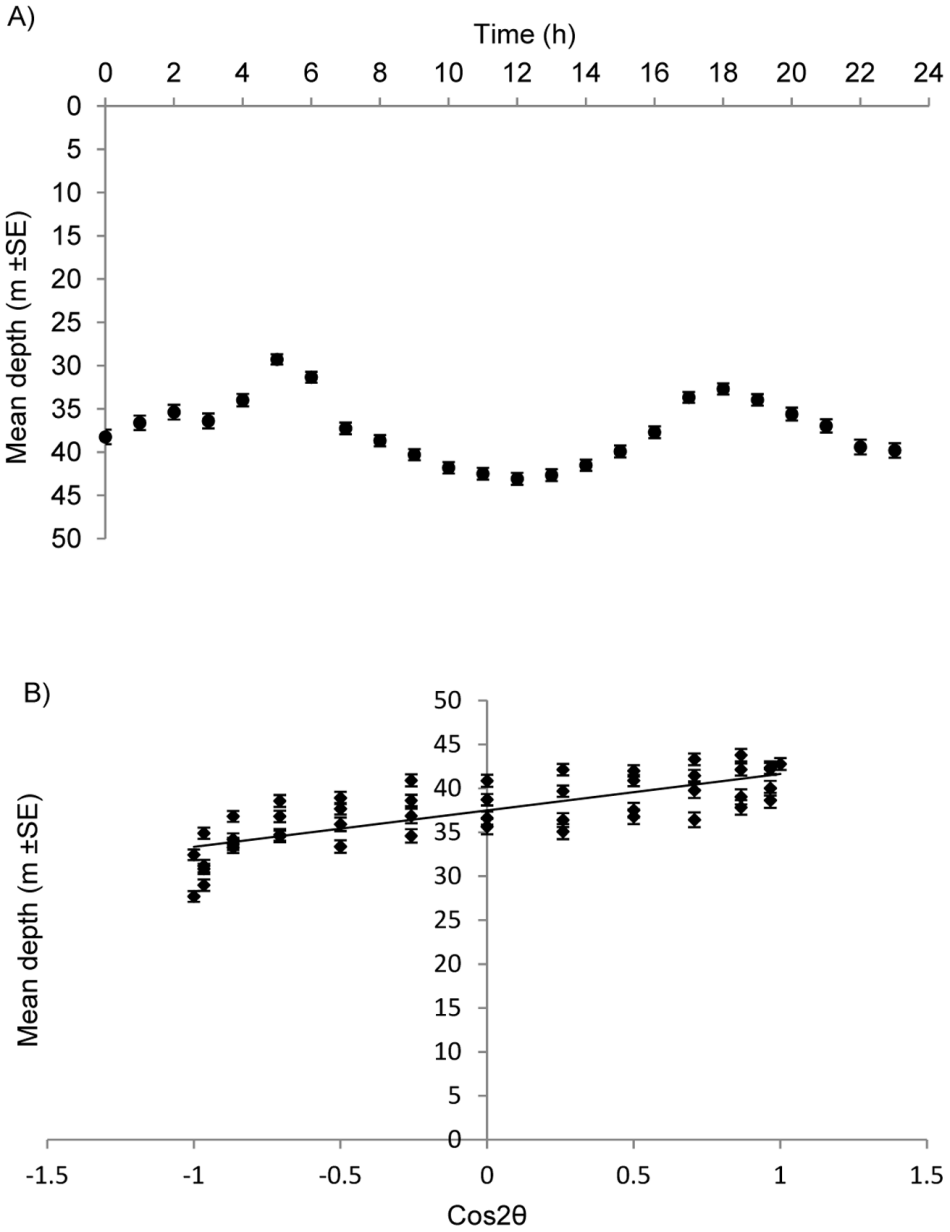
Daily pattern of vertical movements by grey reef sharks in Palau. A) Mean hourly depth of grey reef sharks combined. B) Linear regression of mean depth of grey reef sharks combined as a function of Cos2θ-transformed hours. y = 4.15x + 37.49, R^2^ = 0.59.

**Figure 7:**
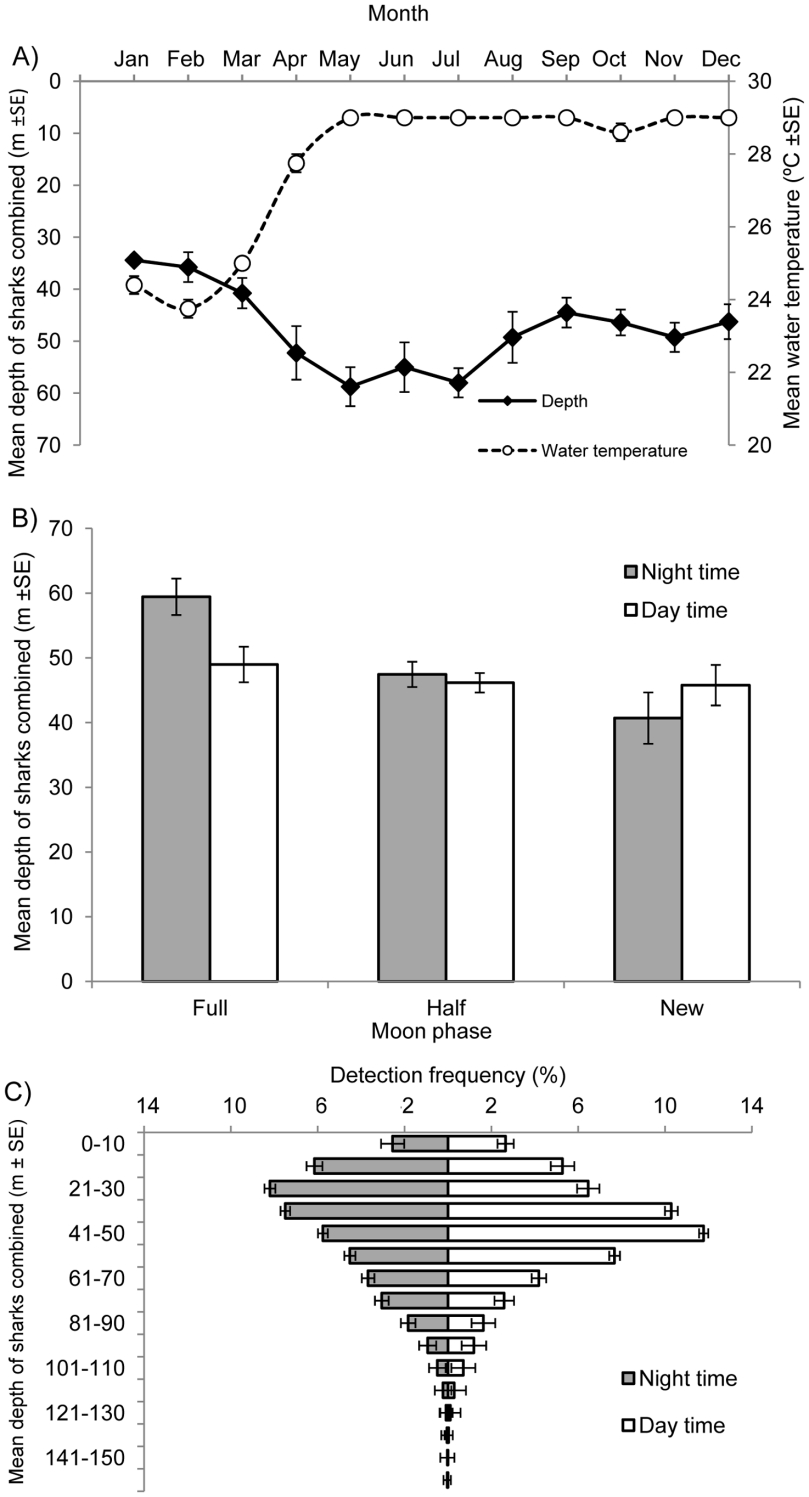
Relationship of depth use by grey reef sharks and environmental variables in Palau in 2010. A) Mean monthly depth of grey reef sharks in Palau and mean monthly water temperature at 57 metres B) Mean depth of sharks in a given moon phase C) Detection frequencies of sharks throughout the water column during the day and night.

The circular regression revealed a cyclical pattern of depth usage on a daily basis (R^2^ = 0.59, p<0.01) (Figure 6). Sharks used shallower waters of around 30 m during dawn (5–6 am) and dusk (6 pm). After sunrise, mean depth gradually increased throughout the morning until noon, when mean hourly depth reached its maximum (∼45 m). Mean depth then declined until sunset. A similar, but less pronounced pattern of depth usage occurred at night (Figure 6). Overall, there was a tendency for the sharks to use shallower waters during the night (Figure 7).

GLMs identified water temperature, lunar phase and the interaction between these variables as the strongest influences on patterns in vertical movement of sharks (*_w_*AIC_c_ = 0.51). These two factors and their interaction explained 60.5% of the deviance in the data set (Table 5) with temperature having the greatest effect on the mean depth of sharks, explaining 43.0% of the deviance. Water temperature (measured at 57 m) was lowest from January to March when it ranged from 23–25°C. Temperatures then increased to ∼29°C and remained constant throughout the remainder of the year. The lower water temperatures in January coincided with use of the shallowest mean depths by sharks. As water temperatures increased at 57 m, sharks occupied deeper waters, averaging 55 m depth from April to August (Figure 7). Although there was little change in water temperature from August to December, sharks tended to occupy shallower habitats (mean 45 m depth) at this time.

**Table 5:** Generalised Linear Model ranking results of the average depth of tagged grey reef sharks (with depth sensors) in 2010 (response variable) versus the effect of lunar phase (Moon) and water temperature at 57 metres (Temperature). Models compared based on Akaike's Information Criteria corrected for small samples (AICc).

Model	LL	df	AICc	dA ICc	wA ICc	%DE
Depth ∼1 (Null)	−196. 464	1	392. 928	62. 438	0. 0000	0.0
Depth ∼ Moon	−186. 767	2	373. 535	43. 044	0. 0000	18.8
Depth ∼ Temperature	−174. 273	2	348. 547	18. 056	0. 0001	43.0
Depth ∼ Temperature + Moon	−165. 278	3	330. 556	0. 066	0. 4917	60.4
**Depth ∼ Temperature** * Moon	−**165.** **245**	**4**	**330.** **491**	**0.** **000**	**0.** **5082**	**60.5**

LL: Maximum Log Likelihood, df: degrees of freedom, dAICc: difference of AICc of a given model to the model with best fit, wAICc: AICc weight and %DE: Percentage of deviance explained. Model with best fit highlighted (bold). (*) Interaction between variables.

Lunar phase also influenced the mean depth of sharks. Depths of sharks at night increased from 40 m during the new moon, to 60 m on the full moon (Figure 7). Contrastingly, the mean depth of sharks during the day did not differ with lunar phase, remaining between 45–50 m (Table 5).

## Discussion

### Site fidelity and horizontal movement

Grey reef sharks in Palau displayed high levels of inter-annual residency, with tagged sharks detected at the same sites along the outer reef slopes for over two years. In both northern and southern areas, most grey reef sharks also displayed residency at the scale of single sites (i.e., residency index higher than 0.5 and attendance for more than 12 hours per day). Unsurprisingly, the highest numbers of sharks detected daily were recorded at the sites where the majority were tagged (Blue Corner and Ulong Channel). There was however, some seasonal variation in attendance in both northern and southern areas, with fewer sharks detected during winter and spring than summer months.

Our results are consistent with those of Field et al. [29] and Barnett et al. [1] who also found strong patterns of site fidelity of grey reef sharks at the remote offshore atolls of the Rowley Shoals (17°19′S, 119°20′E, 250 km from the north-west coast of Australia) and Osprey Reef (13°54′S, 146°38′E, 143 km off the east coast of Australia), but contrast those of Heupel et al. who found that grey reef sharks displayed relatively low rates of site fidelity on the Great Barrier Reef (GBR, 14°30′S, 145°33′E)[8]. In the latter study, some individuals moved 15–18 km over the monitoring period and were detected on a number of reef platforms. Such differences in the degree of site fidelity of this species could be related to the distribution and connectivity of reef habitats. Heupel et al. [8] noted that the reefs in their array of receivers on the GBR were linked by shallow (20 m depth) passes that may allow easy access for sharks to adjacent reefs. While reef isolation may account for the greater degree of site fidelity of sharks at remote atolls, this does not explain the high degree of site fidelity of grey reef sharks in Palau where sites occurred on a continuous barrier reef that stretched more than 260 km. An additional possibility is that such variation in site fidelity could also be related to the life history traits (for example, sex and maturity) of the tagged animals. At Osprey Reef and in Palau where sharks have a high degree of site fidelity, aggregations of grey reef sharks are almost exclusively composed of females [1] (Meekan et al. unpubl data) and as a result, most animals tagged in both areas were mature females. In contrast, Heupel et al. [8] tagged an equal number of males and females on the GBR. On these reefs females tended to display the strongest patterns of site fidelity, with three of the five tagged females being detected an average of 75% of days during a 150 day monitoring period. In contrast, three of five tagged males were never detected or only monitored for short periods of less than 30 days before disappearing from the study area. The two remaining males were monitored over relatively long times (154 and 167 d) but were only detected on one and 22 (13%) days respectively. Furthermore, the largest movement recorded by their study was undertaken by a male shark that travelled 134 km between atolls in the Coral Sea and the GBR. These results suggest that there may be sex-biased patterns of dispersal and site fidelity in grey reef sharks, a phenomenon that has been recorded in a number of other species, including the shortfin mako (*I. oxyrinchus*), blue (*Prionace glauca*) and hammerhead (*Sphyrna lewini*) sharks [39], [40]. Testing this hypothesis will require the tagging of greater numbers of male sharks, which is likely to be a challenge in locations such as Palau where aggregations are dominated by females.

The description of movement and patterns of attendance by acoustic telemetry studies is typically limited by the number and range of the array of receivers that are deployed to track the subject animals. For species such as sharks that are capable of moving large distances, this frequently results in long periods of absence, when tagged animals remain out of range or away from the monitored areas [3], [8], [29], [41]. These issues need to be considered when tracking data are used to make assertions regarding home range, use of habitat and connectivity. Our tagged sharks displayed high levels of site fidelity and residency throughout the year, implying that our results are robust despite the limited number of receivers in our array. However, there was some degree of variation in site fidelity of several mature females, which is supported by the observation of movements between the northern and southern areas (a distance of 17.2 km) by three females and the extended period of absence (one year) of a shark from the acoustic array. Although the spatial scale of these movements is consistent with results from studies of grey reef sharks on the GBR [8], in the Coral Sea [1] and earlier work on other Micronesian atolls [42] that used an active tracking approach, the limited number of receivers that we deployed means that we may have underestimated the frequency and extent of such movements of tagged sharks. Further expansion of the array of receivers should allow the analysis of fine scale movements of sharks.

### Vertical movement and environmental influences

Grey reef sharks displayed diel patterns of vertical movements. The shallowest depths (30 m) were occupied at dawn and dusk, with sharks using progressively deeper waters until noon. An opposite pattern occurred in the afternoon with sharks gradually ascending until dusk. This cyclical pattern of descent and ascent was less pronounced at night. Other studies have shown that grey reef sharks show crepuscular patterns, possibly caused by foraging behaviour [1], thus ascents to shallow reef areas at dawn and dusk in Palau may also be associated with feeding. Crepuscular patterns of vertical movement associated with foraging behaviour are common in many pelagic sharks including shortfin mako, big eye thresher (*Alopias superciliosus*), school (*Galeorhinus galeus*) and megamouth (*Megachasma pelagicos*) sharks [14], [43], [44], [45]. This behaviour has been associated with the daily vertical movement of prey items [46]. Crepuscular behaviour might also be explained by the active attempts of some species to maintain a preferred isolume [45].

Sharks attained greatest mean depths at midday when sunlight penetrates the water column with minimal reflection and they descended or ascended during the morning and afternoon when reflection at the water surface was greatest. These fine-scale patterns of vertical movement suggest that luminosity might influence the vertical movements of grey reef sharks. Such behaviour has been observed in pelagic sharks, including the megamouth [45], although it is thought to occur over a much greater range of depths (around 100 m) than observed in grey reef sharks (15 m). Archival tags that record both depth and light levels could provide insights into role of luminosity in the vertical distribution of reef sharks.

There were also distinct seasonal patterns of depth use by grey reef sharks in Palau. In winter (January and February), when water temperatures at 60 m attained seasonal lows (23–25°C), sharks tended to utilise shallow waters (mean monthly depths of ∼35 m). A steady increase in water temperature at the end of winter and spring (March to May) and displacement of the thermocline to waters below 60 m [22] was paralleled by an increase in the range of depths used by sharks from 40 to 60 m. Temperature shifts in the order of 1°C to 4°C are generally enough to produce major responses in fish behaviour and distribution [47] and water temperature is an important environmental parameter for grey reef sharks (and many other species of shark) since they can display behavioural strategies that function to maintain optimum body temperature [7], [11], [17], [18]. In Palau, the shallow water (<15 m) temperatures on the outer reef tend to remain relatively constant throughout the year, while deeper waters (>60 m) may vary by as much as 10°C between seasons [22]. The seasonal pattern of vertical movement observed in our study suggests that in winter, the optimum thermal habitat of grey reef sharks might be restricted to a smaller surface layer of the water column. Many other sharks are known to display vertical movements driven by thermal preferences and this behaviour has been recorded in laminids including shortfin makos and white (*Carcharodon carcharias*) sharks. These regularly descend to the thermocline to feed, but then return to shallow, warmer waters where they spend the majority of their time [14], [48]. Similarly, there is evidence that whale sharks (*Rhincodon typus*) spend long periods warming up their bodies in the surface after long deep divers in cold waters [49]. There is also extensive evidence that coastal, reef and oceanic sharks also use warm waters for behavioural thermoregulation [7], [17], [50], a strategy that optimises physiological and metabolic processes [18], [51].

At night, the mean depth inhabited by grey reef sharks increased through the lunar cycle, so that the greatest depths coincided with the full moon. Similar patterns recorded by tagging studies of pelagic species such as swordfish, yellowfin and big eye tuna, suggests that such effects of lunar illumination might be widespread among large pelagic predators [13], [15], [16]. Fisheries data for a range of other pelagic sharks and tunas also support this idea, although some species such as the black marlin (*Makaira indica)* show the opposite pattern, with catches increasing in shallow waters during the full moon [52]. Some coastal sharks also display evidence of lunar influences on depth distributions. For example, the nocturnal patterns of vertical migration of school sharks are depressed during the nights of full moon [44], while juvenile white sharks descend to greater depths with higher frequency during the nights of full moon [48]. Given that greater activity patterns of grey reef sharks during twilight and night hours are thought to be related to foraging behaviour [1], [5], [42], it seems likely that the use of deeper waters during the full moon could be a response to equivalent changes in distribution patterns of their prey. In pelagic systems, such reciprocal patterns in distribution of predator and prey species are very common, with cyclical variation in luminosity of the moon driving changes in the depth distribution of mesoplankton at night [46], [53], which in turn influences the depth distribution of their predators [13], [53]. Alternatively, or possibly in addition, the increase in depth shown by grey reef sharks may be an anti-predator response where sharks seek to avoid the conditions of increased light nearer the surface that may aid the hunting abilities of larger sharks, both of their own and other species.

The complexity of coral reef habitats presents a range of technical challenges that need to be addressed for accurate interpretation of acoustic monitoring data [54]. The analysis of the receiver metrics suggested that the mean performance of our receivers was comparable to earlier work on shark movements in Florida [25] and Western Australia [55]. These metrics also showed that the reduction in performance in 2011, followed by partial recovery, was most likely caused by the tagging of additional sharks in March of that year. The increase in collisions of tag transmissions (as a consequence of more tags in the water) increased the rejection coefficient of the receivers, however we noticed no obvious effects in attendance of sharks that could be attributed to this event. We also observed a drastic decrease of the detection coefficient of the receivers within 200 m, which indicates a relatively short range of detections. We conducted the range testing of the receivers shortly after the tagging event of 2011 and we suspect that the low detection coefficient of the receivers at this time could be partially explained by the collision of tag transmissions due to the increase in numbers of tags in the water. Previous studies of receiver performance indicate that detection ranges in coral reefs environments tend to be low (in the order of a few tens of metres) due to the structural complexity of the habitat [54]. Despite such problems, the very high number of detections (2.3 million) and consistent shark attendance metrics indicated that our results for patterns of site fidelity were not compromised by the technical limitations of acoustic monitoring.

In summary, our study provides the first long-term view of the vertical movements of grey reef sharks within a coral reef environment. Our results confirm previous suggestions that grey reef sharks display strong levels of site fidelity that persist across years, at least for some components of the population. Patterns of daily attendance of sites and vertical movements varied on diel and seasonal cycles. Diel and lunar changes in vertical movement patterns were possibly related to foraging, while seasonally, sharks avoided cooler water temperatures at depth during winter. A better understanding of the role of sharks in coral reef ecosystems now requires integration of such observations into the development of models of the physiology and behavioural ecology of reef sharks.

## Supporting Information

Figure S1 Timeline of acoustic receiver operation in Palau. Plot indicates functioning period (x-axis) of each receiver (y-axis), US  = Ulong Sand Bar, UC =  Ulong Channel, SC =  Siaes Corner, ND out =  New Drop-off Outgoing, ND in =  New Drop-off Incoming, BC out =  Blue Corner Outgoing and BC in =  Blue Corner Incoming. Arrows indicate download events.
(TIF)

Figure S2 Metrics of receiver performance during grey reef shark acoustic monitoring period in Palau. Graphs describe the Detection efficiency (top) and Rejection coefficient (bottom) of receivers in the northern (left) and southern area (right) of the study site.
(TIF)
