# Supplementary figures and images for: Environmental Influences on Patterns of Vertical Movement and Site Fidelity of Grey Reef Sharks (Carcharhinus amblyrhynchos) at Aggregation Sites
Source: PLoS One. 2013 Apr 10;8(4):e60331. doi: 10.1371/journal.pone.0060331 (PMC3622676; doi:10.1371/journal.pone.0060331)

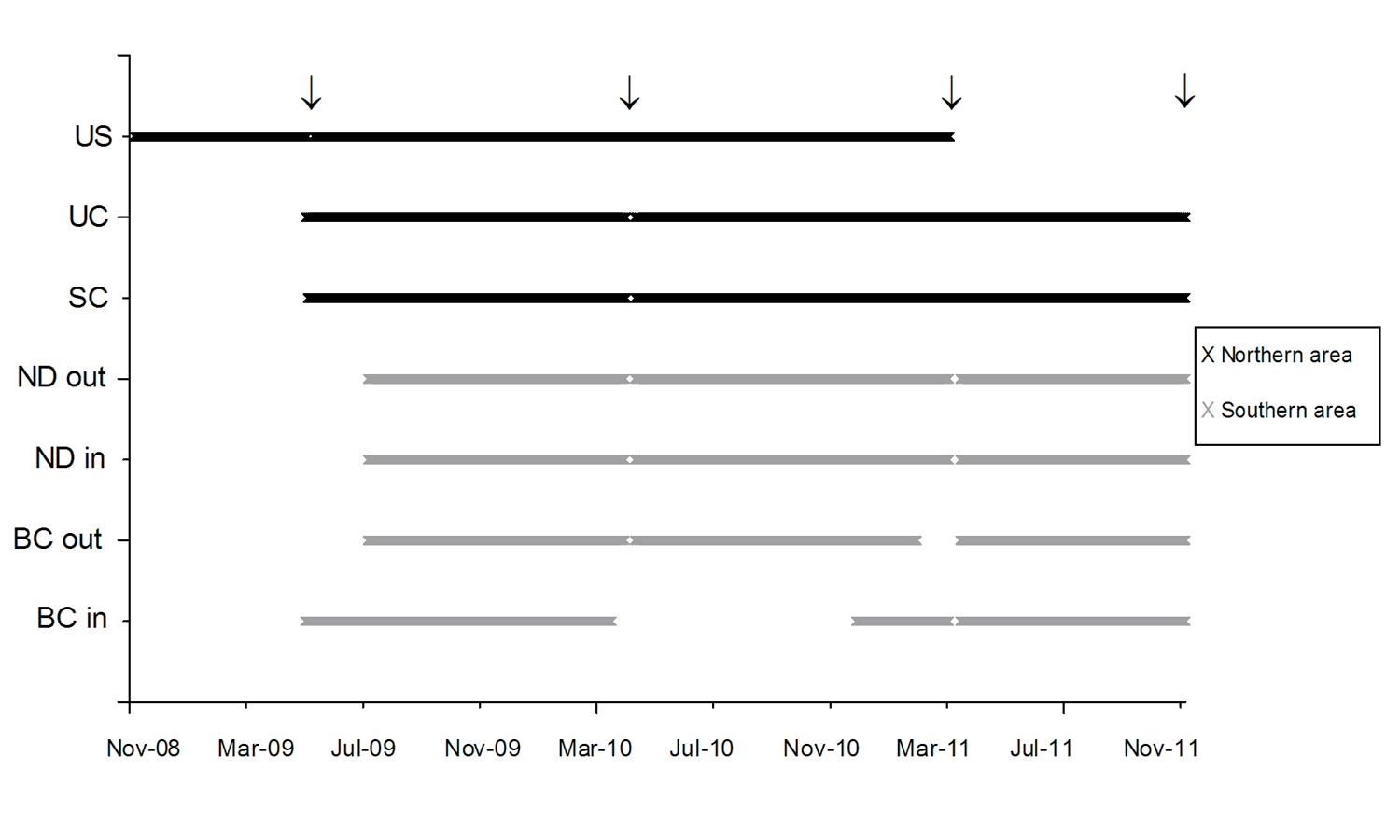

Supplement: Figure S1 — Timeline of acoustic receiver operation in Palau. Plot indicates functioning period (x-axis) of each receiver (y-axis), US = Ulong Sand Bar, UC = Ulong Channel, SC = Siaes Corner, ND out = New Drop-off Outgoing, ND in = New Drop-off Incoming, BC out = Blue Corner Outgoing and BC in = Blue Corner Incoming. Arrows indicate download events. (TIF) [file pone.0060331.s001.tif]

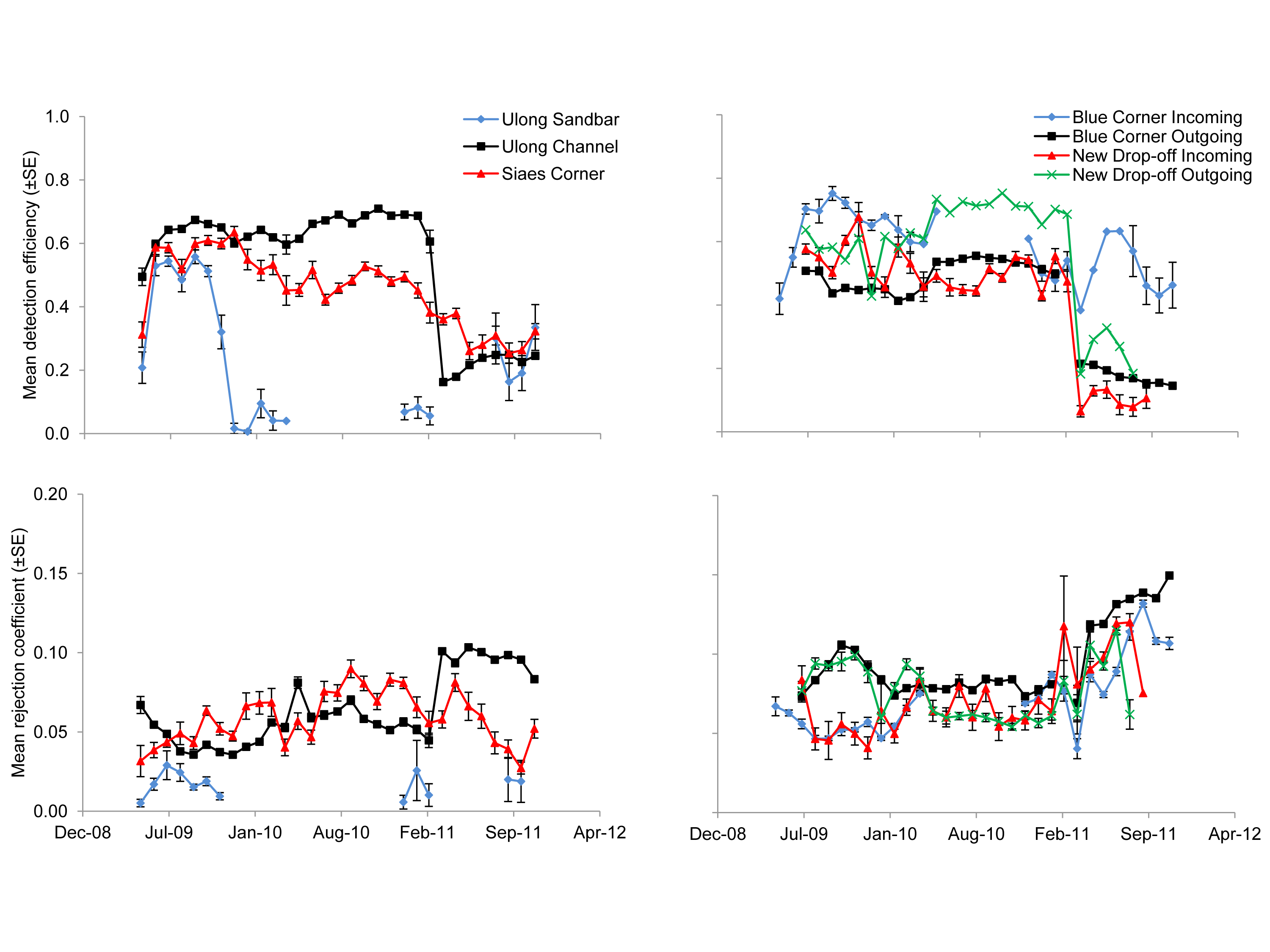

Supplement: Figure S2 — Metrics of receiver performance during grey reef shark acoustic monitoring period in Palau. Graphs describe the Detection efficiency (top) and Rejection coefficient (bottom) of receivers in the northern (left) and southern area (right) of the study site. (TIF) [file pone.0060331.s002.tif]
